# FLT-PET for early response evaluation of colorectal cancer patients with liver metastases: a prospective study

**DOI:** 10.1186/s13550-017-0302-3

**Published:** 2017-07-10

**Authors:** Marie Benzon Mogensen, Annika Loft, Marianne Aznar, Thomas Axelsen, Ben Vainer, Kell Osterlind, Andreas Kjaer

**Affiliations:** 1 Department of Oncology, Rigshospitalet, University of Copenhagen, Blegdamsvej 9, 2100 Copenhagen, Denmark; 2 Department of Clinical Physiology, Nuclear Medicine & PET and Cluster for Molecular Imaging, Rigshospitalet and University of Copenhagen, Copenhagen, Denmark; 3 Department of Radiology, Rigshospitalet, University of Copenhagen, Copenhagen, Denmark; 4 Department of Pathology, Rigshospitalet, University of Copenhagen, Copenhagen, Denmark

**Keywords:** FLT-PET, Colorectal cancer, Early evaluation, [18 F]-3′-Deoxy-3′-fluorothymidine, Molecular imaging

## Abstract

**Background:**

Fluoro-L-thymidine (FLT) is a positron emission tomography/computed tomography (PET/CT) tracer which reflects proliferative activity in a cancer lesion. The main objective of this prospective explorative study was to evaluate whether FLT-PET can be used for the early evaluation of treatment response in colorectal cancer patients (CRC) with liver metastases. Patients with metastatic CRC having at least one measurable (>1 cm) liver metastasis receiving first-line chemotherapy were included. A FLT-PET/CT scan was performed at baseline and after the first treatment. The maximum and mean standardised uptake values (SUV_max_, SUV_mean_) were measured. After three cycles of chemotherapy, treatment response was assessed by CT scan based on RECIST 1.1.

**Results:**

Thirty-nine consecutive patients were included of which 27 were evaluable. Dropout was mainly due to disease complications. Nineteen patients (70%) had a partial response, seven (26%) had stable disease and one (4%) had progressive disease. A total of 23 patients (85%) had a decrease in FLT uptake following the first treatment. The patient with progressive disease had the highest increase in FLT uptake in SUV_max_. There was no correlation between the response according to RECIST and the early changes in FLT uptake measured as SUV_max_ (*p* = 0.24).

**Conclusions:**

No correlation was found between early changes in FLT uptake after the first cycle of treatment and the response evaluated from subsequent CT scans. It seems unlikely that FLT-PET can be used on its own for the early response evaluation of metastatic CRC.

## Background

Response evaluation during cancer treatment is normally based on the response evaluation criteria in solid tumours (RECIST) looking for tumour shrinkage [1]. However, molecular changes precede tumour shrinkage, and therefore, there is an increased interest in functional imaging in the search for early response evaluation attributes [2, 3].

[^18^F]-3′-Deoxy-3′-fluorothymidine (FLT) is a modified thymidine analogue developed as a positron emission tomography (PET) tracer to reflect proliferation. FLT crosses the cell membrane and becomes phosphorylated by thymidine kinase 1 (TK-1), which is the first enzyme in the salvage pathway of thymidine synthesis as part of the DNA synthesis. FLT is not incorporated into the DNA, but becomes dephosphorylated at a slow rate. Phosphorylation causes FLT to be trapped inside the cell for a relatively long period, making it suitable for tracer imaging [4]. Several small studies have shown a correlation between the uptake of FLT and the histopathological marker of proliferation Ki-67 [5]. It has also been shown that FLT accumulates proportionally with TK-1 [6]. Furthermore, we have previously shown in preclinical studies that FLT is a sensitive and early predictor of morphological therapy response in various cancers [7–10].

Colorectal cancer (CRC) is the third most common type of cancer worldwide [11]. Approximately 20% of patients with newly diagnosed CRC have distant metastases at the time of diagnosis, the most common site being the liver [11]. Moreover, another 50% of CRC patients will develop liver metastases at some time during their disease. The only definitive treatment for metastatic CRC is surgical resection if possible [12]. A revolution in the treatment of metastatic CRC has taken place in the last decade—especially in the surgical field—resulting in more patients being candidates for resection, which has been followed by a significant improvement in the 5-year survival rate of patients resected for liver metastases [12]. It is therefore crucial to identify patients who are not responding as early as possible as an ineffective treatment could jeopardise their chance of being candidates for resection. Furthermore, the benefit of saving patients from unnecessary side effects of a treatment that does not work is also worthwhile in a palliative setting.

Preclinical studies with cell lines and xenografts [4, 13–16] as well as a few clinical studies [17, 18] have suggested the possibility that FLT-PET could be used for the early assessment of treatment response in CRC. To the best of our knowledge, to date, the potential of FLT-PET for the early response evaluation of CRC during chemotherapy has been investigated in only three clinical studies with 2, 12 and 18 patients, respectively [17–19]. Just one of these studies used liver metastases as measurable outcome lesions [17], while none of them used the currently preferred treatment regimen, i.e. addition of a targeting agent to the chemotherapy.

The present study therefore aimed to assess the potential of FLT-PET/computed tomography (CT) in the early response evaluation of CRC based on measurable liver metastases. The early changes in FLT uptake were compared to RECIST after three cycles of treatment. It is acknowledged that RECIST, especially after the introduction of bevacizumab, have limitations in response evaluation in liver metastases since increasing necrosis does not necessarily lead to tumour shrinkage [20, 21]. Hence, it has been suggested that, in combination with volumetric changes, the morphologic changes of liver metastases such as, sharpness of the rim and the heterogeneity of contrast enhancement seen on CT or magnetic resonance (MR) may also be useful for evaluating response. Although morphology is not included in the clinical guidelines, a study by Chun and co-workers found that morphological criteria based on attenuation changes on CT correlated with the pathologic response and was associated to overall survival (OS) [20]. Therefore, in this study, changes in FLT uptake shortly after the initiation of treatment were compared not only to the outcome of RECIST but also to the changes in attenuation of liver lesions on CT after three treatment cycles.

## Methods

### Study design

The study was an explanatory prospective trial to assess whether FLT-PET/CT can predict the response to first-line chemotherapy in CRC with liver metastases early after the initiation of chemotherapy. The primary outcome measure was the standard anatomic response determined using RECIST, version 1.1, applied on CT scans after three treatment cycles. Tumour shrinkage is necessary for resection to be possible in patients with only potentially resectable disease. Therefore, an aggressive approach is especially important in these cases. Tumour shrinkage after three cycles was therefore chosen as the primary endpoint. Furthermore, according to the criteria detailed by Chun et al. [20], a morphologic response assessment was performed based on the changes in attenuation seen on CT after three cycles of treatment.

The study was conducted in accordance with the Helsinki Declaration. All patients provided written consent before participation. The study protocol was approved by the Regional Ethics Committee of The Capital Region of Denmark (ref. no. H-3-2012-041).

### Patient population

Patients referred to the Department of Oncology at Rigshospitalet, Copenhagen University Hospital, for palliative or neoadjuvant treatment for CRC with liver metastases were considered for inclusion in the study. The inclusion criteria were (i) at least one measurable liver metastasis (>1 cm) on the diagnostic CT scan, (ii) histologically confirmed adenocarcinoma in the primary tumour and (iii) a ECOG performance status of 0–1 [22]. This meant that patients with liver only as well as patients with more advanced disease could be included. The patients were required to be chemonaïve or to have completed adjuvant chemotherapy at least 6 months prior to inclusion. Exclusion criteria were (i) acute myocardial infarction within 6 months or unstable angina, (ii) previous allergic reaction to a contrast agent, (iii) systolic blood pressure higher than 150 mmHg and (iv) patients not fit for two-drug cancer treatment. Patients were treated with first-line chemotherapy independently to this study according to local guidelines until disease progression, refusal of treatment, toxicity criteria for discontinuation were met, possibility for surgical resection or 9 months of treatment. The patients were evaluated after every third treatment cycle or whenever progression was suspected clinically.

### RECIST evaluation

Changes seen on CT from baseline to evaluation after three cycles of treatment were evaluated according to RECIST 1.1. According to RECIST, a maximum of five lesions were included in the assessment, hereof maximum two lesions per organ. All had at least one liver lesion included. The primary tumour was not included. Evaluations were performed by a specialised radiologist. According to the guidelines, stable disease (SD) is defined as a decrease in measurement of no more than 30% or an increase less than 20% [1]. Patients having progressive disease (PD) (i.e. increase of largest diameter of more than 20%) or SD were characterised as non-responders, while responders were defined as patients experiencing partial response (PR) or complete response (CR).

### FLT-PET/CT imaging

FLT was routinely synthesised in-house according to standard procedures [23]. Approximately 350 MBq was injected intravenously approximately 60 min prior to scanning in a Siemens Biograph 64 PET/CT scanner (Siemens Health Care, Erlangen, Germany). The baseline scan was performed with intravenous and oral contrast media according to general clinical practice while the follow-up scans were performed as PET/CT with low-dose CT for attenuation correction purposes. The PET acquisition time was 2–5 min per bed position depending on body mass index. The patients were scanned from the skull base to proximal thighs. FLT-PET/CT scans were obtained twice: before onset of the treatment as baseline and after initiation of chemotherapy between day 5 and 15 (mean; day 10).

Visually defined regions of interest were analysed quantitatively on the FLT-PET scans by an experienced nuclear medicine physician blinded to the RECIST outcome on the evaluation scan after three cycles of treatment. The same set of metastatic lesions depicted by the radiologist as target lesions for RECIST was evaluated by FLT-PET. FLT uptake was measured as maximum and mean standardised uptake values (SUV_max_, SUV_mean_). The percentage changes of SUV between baseline and evaluation scans were calculated for all target lesions. Primary tumours were evaluated by both FLT/PET and RECIST, but because of difficulties in performing accurate repeated measures due to mobility of the primary tumour, these measures were not included in the RECIST and FLT/PET comparison.

### Morphologic assessment

Using the criteria detailed by Chun et al. [20], an expert liver radiologist assessed the morphologic changes observed on CT. Each metastasis was assigned to one of three morphology groups where group 1 was defined as metastases with homogeneous low attenuation with a thin, sharply defined tumour-liver interface, and group 3 was characterised by heterogeneous attenuation and a thick, poorly defined tumour-liver interface. Metastases that could not be allocated to these two groups were included in group 2. The metastases were categorised on the baseline CT as well as on the evaluation CT. An optimal response was defined as reallocation of each individual metastasis from group 3 or 2 to group 1. An incomplete response was defined as reallocation from group 3 to group 2. No response characterised metastases not changing groups.

### Statistical analysis

The hypothesis of this study was that early changes in FLT uptake might be predictive for response. The Wilcoxon signed-rank test for paired data was used to assess whether there were changes in FLT uptake measured as SUV from baseline to evaluation. To test for differences in SUV measures between responders and non-responders, the Mann-Whitney *U* test was used. The primary endpoint was tumour shrinkage after three cycles of treatment, and therefore, correlation between early changes in SUV and the RECIST measurements was tested by the Pearson correlation coefficient. A two-sided *p* value less than 0.05 was considered statistically significant. Cox regression analysis was performed to test correlation between early FLT changes (percentage change of FLT) and OS. The positive predictive value for using early increase in FLT uptake as a predictor of patients with PD was calculated based on tumour growth after three cycles of treatment. The statistical analyses were performed with SPSS version 19 software (SPSS Inc., IBM Company, NY, USA).

## Results

### Patient characteristics

**Fig. 1:**
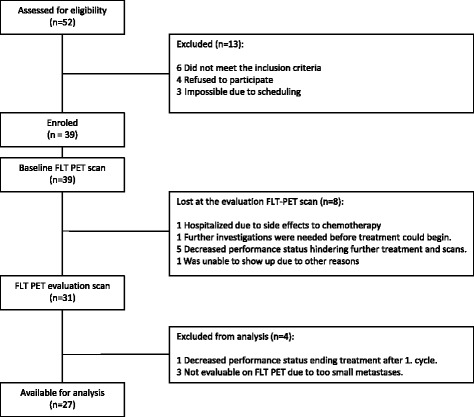
CONSORT flow diagram

Between June 2012 and November 2014, 39 chemonaïve patients were recruited to the study (see the CONSORT flow diagram in Fig. 1). Of these 39 patients, 12 were excluded. Specifically, eight patients did not undergo FLT-PET scanning primarily due to decreased performance status that hindered further treatment, one patient received only one cycle of treatment and three patients’ FLT-PET scans were not evaluable as their metastases were too small. Thus, data from the remaining 27 patients were available for further analysis. Patient characteristics are listed in Table 1. All patients received first-line treatment according to local guidelines. During the study period, guidelines were slightly altered for patients without a mutation in the *RAS* genes (ras-wild-type) from bevacizumab, capecitabine and oxaliplatin (bev-CAPOX) to cetuximab, 5-fluorouracil (5-FU; intravenous) and irinotecan (cet-FOLFIRI), while patients with a *RAS* mutation continued receiving bev-CAPOX. Capecitabine is an oral drug given b.i.d. for 2 weeks while 5-FU is given as a bolus day 1 followed by 46 h on a pump. Bevacizumab, oxaliplatin and irinotecan is given as a bolus day 1.

**Table 1:** Patient characteristics

Characteristics	*n* = 27	Percentage
Gender		
Male	15	56%
Female	12	44%
Primary tumour		
Right	7	26%
Left	12	44%
Rectum	8	30%
Primary tumour		
Resected	11	41%
In situ	16	59%
Metastases		
Only liver	13	48%
Lymph nodes	12	44%
Lung	5	19%
Peritoneal carcinosis	2	7%
Chemonaive	27	100%
RAS status		
Wild-type	13	48%
Mutated	14	52%
Pre-treatment CEA	Mean 279	Range 2–2650 ng/ml
Evaluation CEA	Mean 77	Range 2–899 ng/ml
Median age	64	Range 45–84 years
Treatment		
CAPOX	4	15%
Bev-CAPOX	16	59%
FOLFIRI	1	4%
Cet-FOLFIRI	3	11%
Pan-FOLPOX	3	11%

**Fig. 2:**
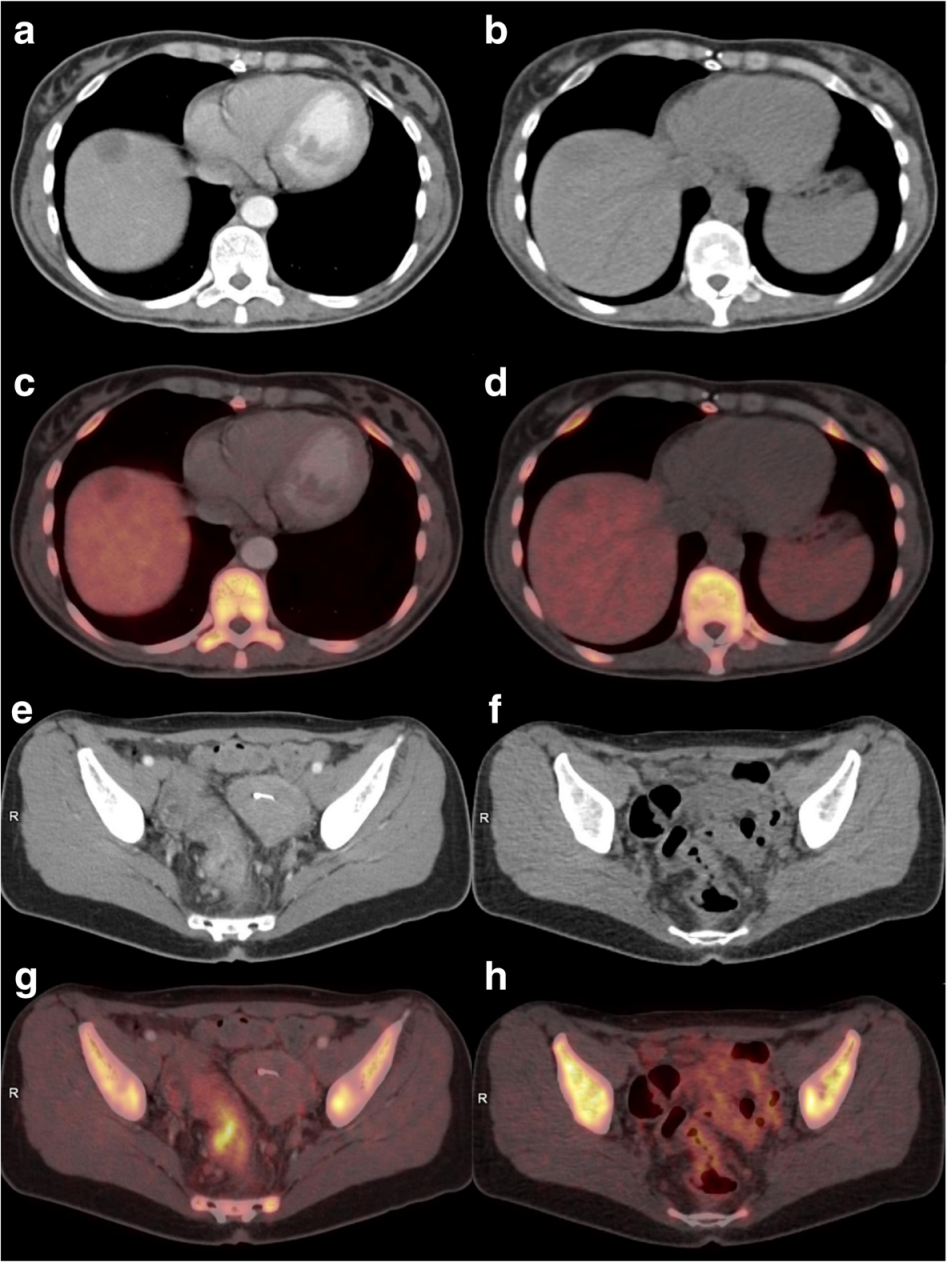
*Left column images* show baseline scanning, and *right column* shows images from the evaluation. **a** A metastasis is seen on the CT as a hypodense focus in the right liver lobe. **c** The corresponding PET/CT reveals slight pathological FLT uptake in the liver metastasis and relatively high physiological FLT uptake in the normal liver parenchyma. **b** The metastasis is more hypodense at the time of evaluation and the corresponding PET/CT. **d** Decreased FLT uptake as a non-active focus in the right liver lobe. **e** The primary tumour in rectum (baseline) is visualised on CT with the corresponding PET/CT. **g** High FLT uptake in the tumour. Physiological high FLT uptake is seen in the bone marrow and in the normal intestine. There is no uptake in the uterus in the left side of the pelvis. **f** At the evaluation, structural shrinkage of the primary tumour on CT is shown and the corresponding PET/CT. **h** Normalised FLT uptake in the residual tumour. There is physiological FLT uptake in the bone marrow and in the small intestine in the left side of the pelvis

The median time interval between the baseline scan and initiation of treatment was 5 days (range 1–8), and between initiation of treatment and FLT/PET evaluation was 10 days (range 5–15). Images from an FLT-PET/CT are shown in Fig. 2.

### FLT vs. RECIST and survival

Based on RECIST 19 patients (70%) experienced PR, seven (26%) had SD and one (4%) had PD after three treatments. The median follow-up time was 27.9 months, in which 12 patients developed PD and ten died. Seven patients had a liver resection after three cycles of treatment, and a further nine patients had a liver resection performed later during their treatment. Follow-up time was between 24.2 and 43.9 months in patients still alive.

**Fig. 3:**
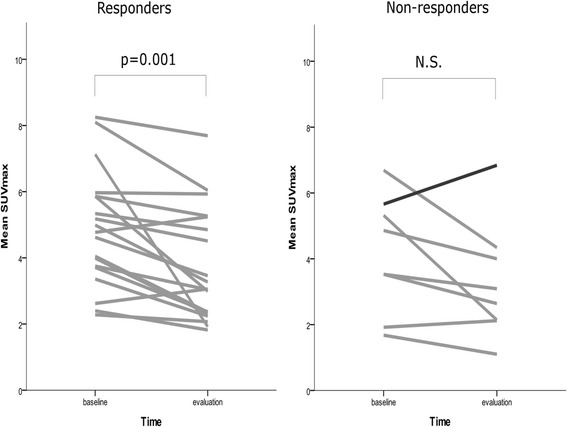
Changes in FLT uptake from baseline to the day of early evaluation. SUV_max_ decreased significantly in the group of responders, whereas the group of non-responders did not show a significant decrease in FLT uptake. The patient with progressive disease is illustrated with a *black line* in the group of non-responders

A decrease in FLT uptake measured by SUV_max_ and SUV_mean_ was seen in 23 of the 27 patients (85%), with an equally significant median change of –25% (SUV_max_, *p* = 0.001; SUV_mean_, *p* = 0.005). In the group of non-responders, no significant change was seen from baseline to early evaluation (SUV_max_, *p* = 0.12; SUV_mean_, *p* = 0.12). Division of the patients into responders and non-responders showing their changes in FLT uptake is illustrated in Fig. 3.

**Fig. 4:**
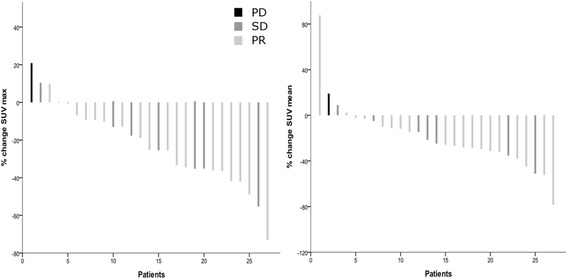
A waterfall plot of FLT-PET response measured as SUV_max_ and SUV_mean_. The percentage change in FLT uptake from baseline to the early response assessment is plotted on the *y*-axis and the patients are listed in order of the amount of change on the *x*-axis. The RECIST characterisations are colour-coded within the bar graph, showing that the patient with progressive disease has the highest increase in FLT uptake, while the patients with stable disease and a partial response are intermingled. *Left panel*: ΔSUV_max_. *Right panel*: ΔSUV_mean_

The patient with PD was the only one without a decrease in lesion size. The maximum FLT uptake (SUV_max_) increased in this patient from 5.67 to 6.84 (21%), which was the highest increase among the patients. Figure 4 illustrates the change in FLT uptake (SUV_max_, SUV_mean_) compared to the RECIST evaluation for all patients. There was no statistically significant correlation between the change in FLT uptake and RECIST outcome (*p* = 0.24) or between the change in FLT uptake in responders versus non-responders (*p* = 0.71). In contrast, the change in carcinoembryonic antigen (CEA) from baseline to evaluation correlated with the percentage change in lesion size (*p* = 0.015) and with RECIST outcome (*p* = 0.047). Analysing all 27 evaluated patients (16 of which had a liver resection), the change in FLT uptake was not related to survival (*p* = 0.79, Wald test, Cox regression analysis). An early increase in FLT uptake (>2%) was a poor predictor of tumour growth (RECIST) after three cycles of treatment as the positive predictive value (PPV) was only 0.25. The negative predictive value (NPV) was 1.

### FLT vs. morphology criteria (Chun)

Application of the criteria of Chun et al. [20] to CT scans distributed the patients into three categories according to observed morphologic changes. Nine patients (33%) had an optimal response, 13 (48%) had an incomplete response and three (11%) had no response. Two patients could not be categorised on the basis of morphological changes. One patient experienced a near-complete response, making an estimation of attenuation difficult, while the other patient was evaluated by MR instead of CT to help the surgical team plan a liver resection.

There was no correlation between the morphological changes after three cycles of treatment [20], and the early changes in FLT uptake, neither measured as SUV_max_ or SUV_mean_ (*p* = 0.62; *p* = 0.94). Likewise, no correlation found between the morphological changes and (i) the RECIST characterisation or (ii) the percent change in tumour size (*p* = 0.79; *p* = 0.92). Four patients were characterised as having an optimal morphological response and yet were classified by RECIST as having SD. Three patients were categorised to have no morphologic response. At CT scan, they had either an increase in tumour size or very modest tumour shrinkage.

## Discussion

Imaging reflecting early cellular changes predicting the response to a given treatment is a non-invasive approach to personalised medicine. The current clinical means of measuring the clinical response by imaging is performed by evaluation using RECIST which is strictly based on changes in tumour size [1]. Since cellular and molecular changes may precede changes in tumour volume, new methods are under investigation [24]. As [18 F]-fluoro-deoxy-glucose (FDG) not only accumulates in tumour tissue but is also seen in inflammatory tissue, new tracers more specific for malignancy are therefore warranted. FLT has been assessed for use in early response evaluation in a few small studies and has been found to be superior to FDG in differentiating tumour from inflammation [25, 26].

To the best of our knowledge, this study is the largest study evaluating FLT-PET/CT for early response assessment of chemotherapy-treated CRC patients with liver metastases. Only one other study has previously evaluated liver metastases as measurable lesions. The study included a mixture of patients with colorectal and breast cancer, and a change in FLT could separate responders from non-responders with a sensitivity of 83% and a specificity of 78% [17]. Other studies have only evaluated extrahepatic lesions. A reason may be that 18-F accumulation is high in normal liver tissue because of FLT glucuronidation in the hepatocytes [18, 19]. In spite of this obstacle, we found it important to investigate the FLT methodology in liver metastases because they very often are the main target in treatment of CRC. Furthermore, liver metastases are more suitable for accurate repeated measurements in contrast to the primary tumour which may be mobile and therefore difficult to capture in the same position from scan to scan. A significant decrease in FLT uptake was seen in the group of responders compared to non-responders; however, this must be interpreted cautiously due to the small sample size in the non-responders’ group. In this patient cohort, looking at the patients individually, we could not separate responders from non-responders based on the changes in FLT uptake.

It can be argued that it is always meaningful to characterise SD according to RECIST as non-responders because SD may represent lesions decreasing up to 30% [1]. For the same reason, instead of forcing data to fit four bins, it has become more and more common to study treatment outcome in waterfall plots since changes in tumour size occur as a continuum [27]. In common clinical practice, SD suggests that the current treatment regimen is continued since this policy has shown superior survival data in metastatic CRC [28, 29]. In this study, all patients with SD had a decrease in lesion size, meaning that the treatment had a beneficial effect. The key point of early response evaluation is to characterise true non-responders so that the current treatment can be discontinued. This is especially important for those patients with potentially resectable disease where an aggressive approach is necessary to achieve resectability. Hence, those patients with “true” PD need to be identified. In this study, the one patient with PD stood out as having the highest increase in FLT uptake; however, no conclusion can be drawn from a single patient with PD.

The majority of the patients in this study received a molecular targeted treatment (70%), in most cases bevacizumab. Bevacizumab is an anti-angiogenic drug that causes morphological changes with a decrease in vascularity followed by a decrease in attenuation and enhancement at imaging even though no changes in tumour size are necessarily seen. It can therefore be debated whether it is reasonable to compare FLT/PET to CT using RECIST because these criteria are often found to be inadequate when bevacizumab is part of the treatment [20, 21]. The fact that many liver metastases resected after neoadjuvant chemotherapy may exhibit a considerable degree of necrosis even though no shrinkage has been proven prior to resection indicates that changes in tumour size do not always reflect the pathologic response [30]. A study by Chun et al. [20] evaluated the treatment response in CRC liver metastases treated with chemotherapy, including bevacizumab. They found that both the morphological changes and RECIST outcome correlated with the histological evaluation of resected metastases, focusing on vital tumour cells, but prediction of minor pathological response was significantly better by morphological changes compared to RECIST. They also demonstrated a correlation between the morphological changes after three cycles of treatment and survival; in contrast, no correlation was seen with RECIST. There are no previous studies on FLT-PET for response assessment during treatment with bevacizumab in CRC. However, a preclinical study investigated the effect of bevacizumab on orthotopic glioblastoma xenografts and found no changes in FLT when bevacizumab was administered as monotherapy [31]. Four of the patients included in the present study were characterised as having SD but showed a good morphologic response, reflecting the inadequacy of RECIST for response evaluation of targeted treatments.

Even though this is the largest FLT/PET study performed on humans with CRC liver metastases, the sample size is still relatively small with too few patients with PD. Another limitation is the variation of treatment regimens even though this reflects the everyday clinical situation. It can be debated whether correlation of the early changes in proliferation measured by changes in FLT uptake with a RECIST assessment of tumour shrinkage or a CT morphologic response is best. Our analysis of changes in FLT uptake and survival was hampered by the fact that a majority of the patients subsequently had a liver resection and thereby were potentially cured. In the ideal setting, all patients should have a liver resection after three cycles of treatment, thus making it possible to correlate changes in FLT uptake to the histological tumour regression, or if no patients were liver resected, it would be possible to correlate FLT uptake changes to the entire cohort’s progression-free survival or OS. In our study, a rather large fraction of the patients (16 out of 27) did undergo a liver resection, thereby making it impossible to correlate early changes in FLT uptake to progression-free survival. It is difficult to predict who will become resectable for their liver metastases following neoadjuvant treatment.

Earlier studies have raised concern about the use of FLT for early response assessment due to the impact of the drugs given on the de novo and salvage pathway of thymidine synthesis. 5-FU inhibits thymidylate synthase and consequently inhibits the de novo pathway following a depletion of the thymidine pool necessary for DNA synthesis. It is known that 5-FU gives rise to an increase in FLT uptake shortly after the drug is given, termed the FLT flare. This flare has been explained by a redistribution of the transporter from the intracellular compartments to the cell surface with no changes in the level of ATP and TK-1 [32], meaning that FLT reaches the cell but is not phosphorylated and therefore not trapped inside the cell. Previous studies have shown that changes in FLT uptake seen after this flare reflect the changes in tumour shrinkage when treated with 5-FU. In our study, all patients were treated with 5-FU either as an infusion or as the prodrug capecitabine. The evaluation FLT-PET scans in this study were performed considerably later than when the flare is reported to occur [6, 18, 32, 33]; therefore, we do not have any concerns regarding potential influence from the flare.

## Conclusions

In conclusion, we were not able to demonstrate correlation between early changes on FLT-PET following the first cycle of treatment and response on subsequent CT scan, neither when evaluated according to RECIST or morphology criteria (Chun). Thus, FLT-PET does not seem to be an applicable methodology for early response evaluation of mCRC treated with 5-FU based chemotherapy.

## Abbreviations

bev-CAPOX: Bevacizumab, capecitabine and oxaliplatin
CEA: Carcinoembryonic antigen
cet-FOLFIRI: Cetuximab, 5-fluorouracil and irinotecan
CR: Complete response
CRC: Colorectal cancer
CT: Computed tomography
FDG: [18 F]-Fluoro-deoxy-glucose
FLT: [18 F]-3′-Deoxy-3′-fluorothymidine
MR: Magnetic resonance
NPV: Negative predictive value
PD: Progressive disease
PET: Positron emission tomography
PPV: Positive predictive value
PR: Partial response
RECIST: Response evaluation criteria in solid tumours
SD: Stable disease
SUV_max_: Maximum standardised uptake values
SUV_mean_: Mean standardised uptake values
TK-1: Thymidine kinase 1
